# The risk of shoulder pain after laparoscopic surgery for infertility is higher in thin patients

**DOI:** 10.1038/s41598-021-92762-3

**Published:** 2021-06-28

**Authors:** Xin You Li, Ming Tian, Ai Zhi Li, Chun Lei Han, Ke Zhong Li

**Affiliations:** 1 Department of Anesthesiology, Yantai Affiliated Hospital of Binzhou Medical University, Yantai, 264100 Shandong People’s Republic of China; 2 Department of Anesthesiology, School of Medicine, Shandong University, 44 Wenhua West Road, Jinan, 250012 Shandong People’s Republic of China; 3 Department of Anesthesiology, The Affiliated Yantai Yuhuangding Hospital of Qingdao University, Yantai, 264000 Shandong People’s Republic of China; 4 School of Public Health and Management, Binzhou Medical University, Yantai, 264003 Shandong People’s Republic of China

**Keywords:** Anatomy, Health care, Medical research, Risk factors

## Abstract

Postlaparoscopic shoulder pain (PLSP) is a common clinical problem that needs to be addressed by medical professionals who are currently perform laparoscopic surgeries. The purpose of this study was to determine the perioperative clinical factors and demographic characteristics associated with PLSP. A prospective observational study was performed with 442 inpatients undergoing laparoscopic surgery for infertility. The pain visual analogue scale was used as the measuring instrument. To identify the predictors of PLSP, we performed multivariate conditional logistic regression. PLSP was correlated with body mass index (BMI, odds ratio = 0.815). The incidence of shoulder pain and more severe shoulder pain in patients with a lower BMI was significantly higher than it was in patients with a higher BMI, and BMI was significantly negatively correlated with PLSP. Most of the patients (95%) began to experience shoulder pain on the first postoperative day, and it rarely occurred on the day of surgery. Patients with lower BMI presented a higher risk of reporting shoulder pain on the first postoperative day. We should identify high-risk patients in advance and make specific treatment plans according to the characteristics of their symptoms.

## Introduction

Laparoscopic-assisted surgery has largely replaced traditional open surgery^1–3^. The main reason is that laparoscopic-assisted surgery has the advantages of decreased postoperative pain^4–8^, earlier return of bowel function, lower morbidity and mortality, quicker postoperative recovery and early hospital discharge^9–11^.

In China, an increasing number of patients have chosen laparoscopic-assisted surgery for infertility assessment and treatment for various reasons in recent years. For patients with infertility undergoing laparoscopic-assisted surgery, the procedure aims to diagnose and solve specific causes of infertility. Most patients will be able to undergo pregnancy and childbirth after surgery. Therefore, in addition to the advantages mentioned above, the choice of laparoscopic-assisted surgery is largely due to the small abdominal incision, minimal scarring and the low incidence of abdominal adhesions, which is conducive to the subsequent pregnancy and delivery process.

Although the abdominal incision is small, unfortunately, many patients complain of postlaparoscopic shoulder pain (PLSP)^12–16^. The reported incidence of PLSP varies (30%-90%) with different surgical methods and studies. Because there are so many patients undergoing laparoscopic surgery every year, even if we estimate the minimum rate of 30%, the number of patients with this clinical problem is quite alarming^17^. Similar to other types of laparoscopic surgery, PLSP is a problem that cannot be ignored in infertility patients after laparoscopic-assisted surgery^18–20^.

According to previous studies, PLSP can last up to 7 days, and sometimes more than 5 weeks^12^. In many cases, the degree of shoulder pain is far greater than that of the incision and internal organs. Most importantly, 72% of patients did not take any opioids to relieve their shoulder pain^15^. It has also been found that PLSP is less responsive to treatment than incision and visceral pain^21^. If we do not give effective treatment immediately, continuous pain will not only increase the discomfort of the patients but also may lead to an increase in the incidence of various postoperative complications and delayed rehabilitation, which will significantly increase the cost of care^14,17^. Furthermore, continuous pain will strongly reduce the satisfaction of the patients. All of these effects are contrary to the original intentions of performing laparoscopic assisted surgery. Therefore, PLSP is increasingly being recognized as a serious clinical problem.

The precise mechanism of PLSP has remained unclear until now. At present, most scholars believe that PLSP is associated with referred pain. The central diaphragmatic pleura and the mediastinal pleura are supplied by phrenic innervation (C3-5), while the supraclavicular nerve (C3-4) conducts sensory input from the acromium process. Pain occurs in the neck or scapula when the phrenic nerve is irritated^16,17,22^.

Many intervention clinical studies aimed at solving PLSP have been conducted^24–32^. Unfortunately, these intervention studies have often found quite varied and sometimes even conflicting results regarding the effectiveness of these interventions, including using drainage^19,32–34^, intraperitoneal instillation of local anesthetics^13,35–37^, pulmonary recruitment manoeuvre^38–40^, gasless laparoscopy^41–43^, warm and humidified dioxide^44–46^, low-pressure pneumoperitoneum^30,31,47^, intraperitoneal normal saline infusion^48,49^ and drugs preventatively or therapeutically^20,50,51^. However, few studies have attempted to identify the risk factors for PLSP^52^. The purpose of this study was to determine the risk factors for PLSP through observation.

## Materials and methods

This study was conducted at the Affiliated Yantai Yuhuangding Hospital of Qingdao University from March 2016 to October 2017. It was approved by the clinical trial ethics committee of Yantai Yuhuangding Hospital of Qingdao University (2016–11) and was registered with the Chinese Clinical Trial Registry (ChiCTR-OOC-16008044). This clinical trial is consistent with the ethical principles of the Helsinki declaration. All patients volunteered to participate in the study and signed written informed consent before the trial.

A total of 442 hospitalized patients undergoing elective laparoscopic surgery for infertility were included in this study. The exclusion criteria were chronic pain syndromes such as fibromyalgia or neck and shoulder pain, a history of long-term use of daily opioids, allergies to medications used in this study such as fentanyl, propofol, and midazolam, impaired cognitive function or inability to understand the study protocol, communication barriers, unstable cardiovascular disease and hypertension, central nervous system disease, endocrine system diseases, and liver or kidney dysfunction.

During the preoperative visit, basic patient information was collected and recorded. The information included the patient's medical history, age, years of education, height, weight, waist circumference and hip circumference. Body mass index (BMI), waist-to-hip ratio, and waist-to-height ratio were calculated.

All patients received similar general anaesthetic and surgical regimens. No premedication was used. Heart rate, arterial blood pressure, and oxygen saturation were monitored in all patients upon arrival at the anaesthesia room. General anaesthesia was induced with midazolam (0.1 mg/kg), fentanyl (4 μg/kg) and propofol (1–2 mg/kg). Cisatracurium infusion (0.15 mg/kg) was used to facilitate tracheal intubation and obtain intraoperative muscle relaxation. Anaesthesia was maintained with oxygen in air (1:2), sevoflurane, propofol and remifentanil. All patients were ventilated with a volume-controlled ventilator (VT10 ml/kg, RR 12/min). The airway plateau pressure, peak airway pressure and CO_2_ pressure in the exhaled air (PetCO_2_) were recorded before and 15 min after the establishment of artificial pneumoperitoneum. Then, tidal volume and respiratory rate were adjusted according to the airway pressure and PetCO_2_. Ondansetron (8 mg) was administered intravenously by the anaesthesiologists to minimize postoperative nausea and vomiting when the surgeons began to close the umbilical trocar sites. Neuromuscular relaxation was reversed pharmacologically at the end of surgery with atropine and neostigmine.

All patients were placed in the lithotomy and Trendelenburg positions during the operation. Laparoscopy was performed with abdominal insufflation of CO_2_ at 12 mmHg using a standard automated insufflator^30,31^. The insufflation gas was not heated or humidified with additional devices^44–46^. All operations were conducted by experienced laparoscopic surgeons (more than 10 years in laparoscopic surgery, involving more than 200 laparoscopic operations) using the standard technique with one 10-mm and two 5-mm trocars. CO_2_ was evacuated at the end of the procedure by manual compression of the abdomen with open trocars^21^. All patients were kept for observation in the PACU until their condition stabilized before moving them to their designated wards.

The following prophylactic analgesic standard treatment was used: intravenous propacetamol (1 g) was used approximately 20 min before the end of surgery, and either intravenous pentazocine (30 mg in the PACU) or oral ibuprofen sustained release capsules (300 mg in the ward) were administered on demand.

### Assessment of pain

The degree of pain was assessed at 6 h, 12 h, 24 h, 48 h and 72 h postoperatively before the patients left the PACU by a 10-cm visual analogue pain scale (0 = no pain to 10 = unbearable pain). We selected the highest value of shoulder pain score at each time point for statistical analysis. The patients were classified into two groups: the absence of shoulder pain (scores equal to zero) and shoulder pain (scores higher than zero).

### Sample size

The sample size was calculated on the basis of the expected incidence of PLSP derived from previous studies^18–20,32^. The formula was as follows:

$${\text{n}} = \left[ {{\text{u}}_{{\upalpha }} ^{2} \times {\uppi } \times \left( {1 - {\uppi }} \right)} \right]/{\updelta }^{2} ,$$

where n: estimated sample size; π: expected prevalence of PLSP, 60% was used; δ: allowing a double-sided type I error rate within 5%, δ = 0.05; α = 0.05, u_α_ = 1.96.

The estimated sample size for the survey was 368. An additional 20% of patients were recruited to overcome dropouts, refusals and exclusions. The study was completed with 442 patients.

### Statistical analysis

The patients were divided into shoulder pain group and absence shoulder pain group. Independent sample t test and Mann Whitney U test were used to compare the differences between the two groups. In order to evaluate the impact of each variable on PLSP, we used univariate binary logistic regression analysis. Then, the influencing factors of PLSP were included in the positive stepwise multiple regression analysis. The patients were divided into four groups according to the Asian BMI standard: group A (BMI < 18.5), group B (18.5 ≤ BMI < 23), group C (23 ≤ BMI < 30) and group D (BMI ≥ 30). The highest VAS score of shoulder pain within 72 h after the operation was compared. Independent-samples t-tests and Pearson’s chi-squared tests were used to compare variables between the groups. P < 0.05 was considered statistically significant. SPSS for Windows (version 18.0, SPSS Inc., Chicago, IL, USA) was used for the statistical analyses.

## Results

Fifty patients were excluded from this study. One case was converted to open surgery because of ovarian cancer detected during the operation, two cases underwent an emergency operation because of intra-abdominal haemorrhage after the laparoscopic operation, and 47 cases were treated by cauterization because of endometriosis found in the diaphragm and upper abdomen during the operation. Finally, 392 patients were included in the statistical analysis.

**Figure 1:**
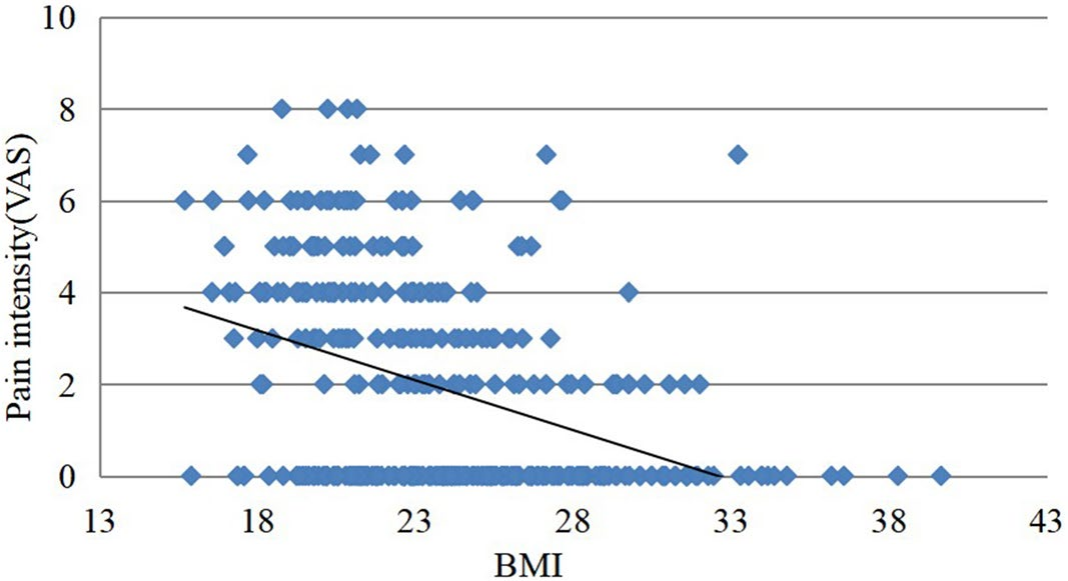
Shoulder pain scores in patients with different BMIs.

**Figure 2:**
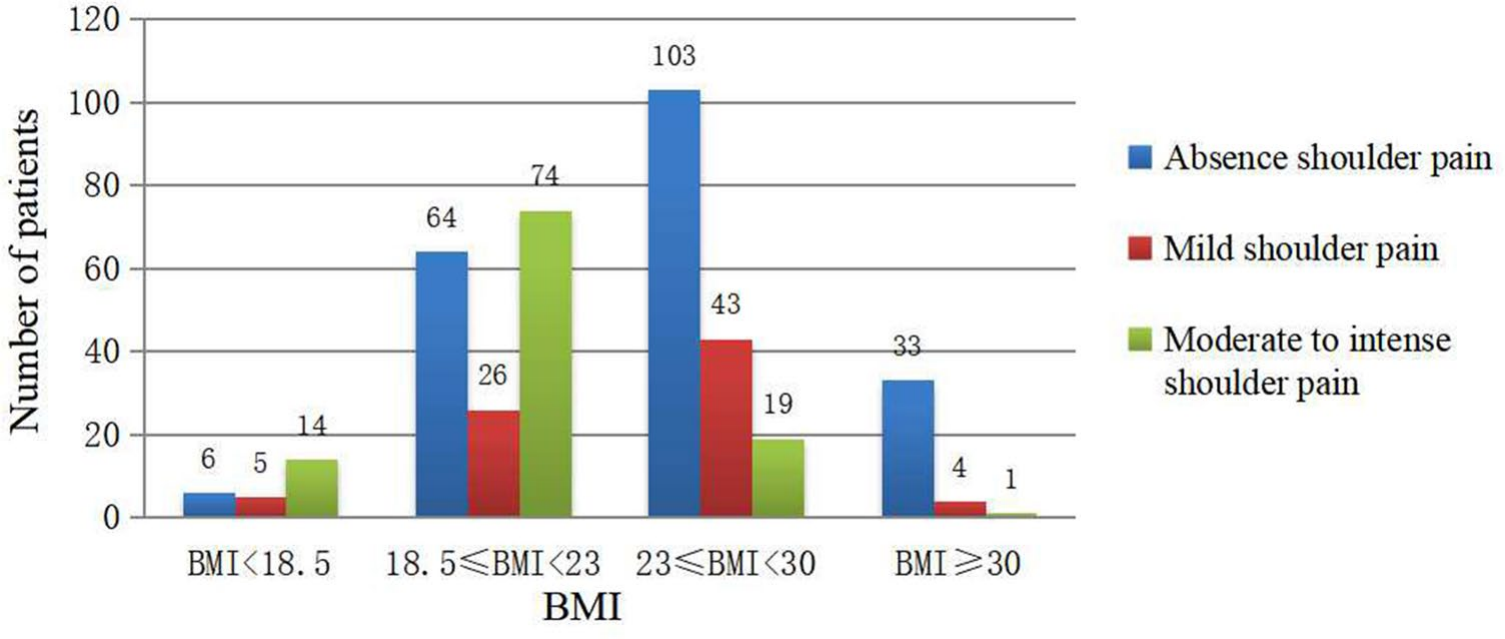
The relationship between PLSP and BMI. The patients were grouped according to the Asian BMI standard. The distribution of the number of people with different degrees of shoulder pain in each group.

**Figure 3:**
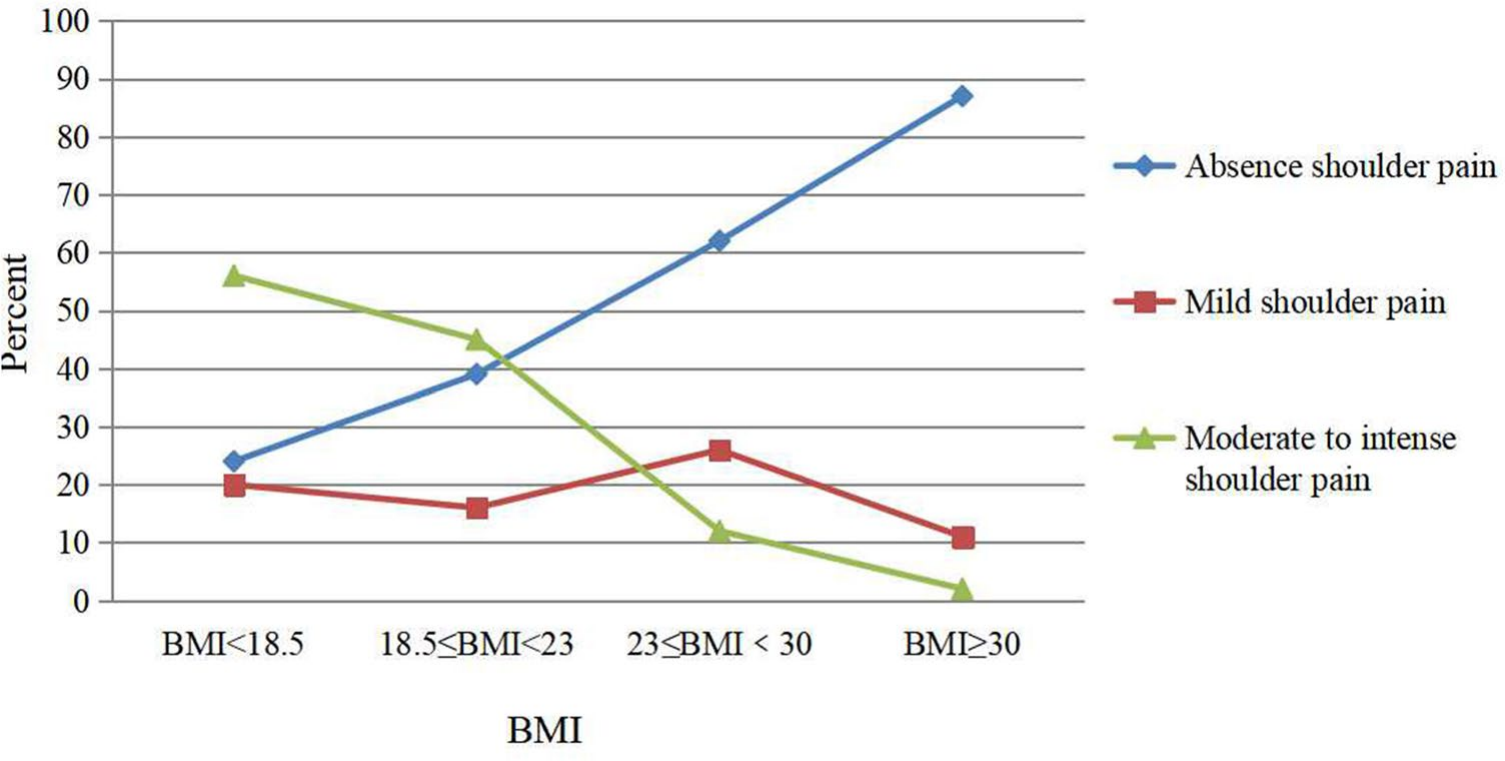
The relationship between PLSP and BMI. The patients were grouped according to the Asian BMI standard. The proportion of patients with different degrees of shoulder pain in each group is shown.

The basic characteristics of the patients’ shoulder pain are shown in Table 1. Nearly half of the patients (186/392, 47.7%) felt unilateral or bilateral shoulder pain during hospitalization. Half of the patients (92/186, 49.4%) had bilateral shoulder pain. The vast majority of patients (95%) began to have shoulder pain on the first day after surgery and rarely on the day of surgery. Table 2 shows that age, height, weight, BMI, waist circumference, hip circumference, waist hip ratio, waist height ratio, peak airway pressure before pneumoperitoneum (P _peak1_), peak airway pressure 15 min after pneumoperitoneum (P _peak2_) and the difference between them (P _peak2_-p _peak1_), airway plateau pressure before pneumoperitoneum (P _platform1_), airway plateau pressure 15 min after pneumoperitoneum (P _platform 2_) and the difference between them (P _peak2_-P _peak1_) of patients have influence on PLSP. However, the number of years in school, duration of surgery, duration of pneumoperitoneum, end-tidal carbon dioxide pressure before pneumoperitoneum (PetCO_2_)_1_ or 15 min after pneumoperitoneum (PetCO_2_)_2_, and the difference between (PetCO_2_)_1_ and (PetCO_2_)_2_ had no effect on PLSP. Stepwise positive regression analysis showed that BMI was an independent predictor of PLSP (OR = 0.815). The scatter plot of BMI and pain score showed that patients with low BMI were more likely to have shoulder pain after operation, and the degree of shoulder pain was also more severe. (Fig. 1). Comparing the highest VAS score of shoulder pain within 72 h after the operation, it was found that there was no significant difference between group A and group B, and there was a significant difference between the other two groups (Table 3). There were significant differences in the incidence of shoulder pain and more severe shoulder pain among the groups. The incidence of shoulder pain and more severe shoulder pain in patients with a lower BMI was significantly higher than it was in patients with a higher BMI, and BMI was significantly negatively correlated with PLSP (Fig. 2, Fig. 3).

**Table 1:** Basic characteristics of the patients’ shoudler pain (n = 392). Values are presented as number or proportion.

	No	Percent (%)	
		/392	/186
Some pain	186	47.4	100
Occasional	56	14.3	30.1
Intermittent	72	18.4	38.7
Constant	58	14.7	31.2
**Site of pain**			
L-shoulder Only	39	9.9	21
R-shoulder Only	55	14	29.6
Both shoulders	92	23.5	49.4
**Quality of shoulder pain**			
Prick/Sharp pain	26	6.6	14
Dull/Distending/Aching pain	160	40.8	86
**Presence of shoulder pain**			
Operative day	6	1.5	3
The first postoperative day	176	44.9	95
The second postoperative day	4	1	2

**Table 2:** Comparison between the two groups and univariate binary logistic regression analysis of the potential influencing factors of LPSP. Values are presented as mean ± standard deviation or median (interquartile range). *P < 0.05.

Variable	Absence shoulder pain (n = 206)	Have shoulder pain (n = 186)	Odds ration	95% CI
Age (years)	31.96 ± 3.56	29.96 ± 3.56	0.940	(0.894–0.988)*
Height (m)	1.62 ± 0.05	1.64 ± 0.04	5059.128	(51.627–495,760.092)*
Weight (kg)	66.63 ± 11.88	60.16 ± 9.53	0.944	(0.925–0.964)*
BMI (kg/m^2^)	25.3 ± 4.33	22.35 ± 3.31	0.815	(0.767–0.866)*
Waist width (cm)	84.89 ± 11.03	78.66 ± 8.32	0.934	(0.913–0.957)*
Hip width (cm)	96.29 ± 8.69	91.78 ± 6.68	0.927	(0.901–0.954)*
Waist/Hip Ratio	0.87 ± 0.05	0.86 ± 0.05	0.000	(0.000–0.005)*
Waist/Height ratio	0.52 ± 0.07	0.48 ± 0.05	0.000	(0.000–0.000)*
Education (years)	11.38 ± 2.9	11.38 ± 2.9	1.053	(0.982–1.129)
Duration of surgery (min)	85(60–115)	85(57.75–120)	0.999	(0.995–1.004)
Duration of pneumoperitoneum (min)	77(52–108)	76.5(15–210)	0.999	(0.995–1.004)
**Plateau airway pressure (P plat)**				
before pneumoperitoneum (P plat_1_)	11.66 ± 2.48	10.44 ± 1.91	0.763	(0.685–0.850)*
15 min after pneumoperitoneum (P plat_2_)	19.7 ± 3.83	17.36 ± 3.1	0.820	(0.768–0.876)*
P plat_2_-P plat_1_	8.04 ± 2.45	6.92 ± 2.15	0.807	(0.736–0.885)*
**Peak airway pressure (P peak)**				
before pneumoperitoneum (P peak_1_)	13.82 ± 3.11	12.21 ± 2.45	0.798	(0.732–0.870)*
15 min after pneumoperitoneum (P peak_2_)	22.01 ± 4.38	19.51 ± 3.65	0.853	(0.807–0.902)*
P peak_2_-P peak_1_	8.2 ± 2.51	7.3 ± 2.2	0.848	(0.776–0.927)*
**Partial pressure of end-tidal carbon dioxide(PetCO**_**2**_**)**				
before pneumoperitoneum (PetCO_2_)_1_	31(29–34)	32(30–34)	1.011	(0.945–1.080)
15 min after pneumoperitoneum (PetCO_2_)_2_	37.5(36–40)	38(36–40)	1	(0.939–1.066)
(PetCO_2_)_2_- (PetCO_2_)_1_	6(5–8)	6(5–8)	0.995	(0.928–1.067)

**Table 3:** Incidence and intensity of shoudler pain between different BMI patients. Values are presented as mean ± standard deviation or number. There was no significant difference between BMI < 18.5 Group and 18.5 ≤ BMI < 23 group. There was significant difference between the other two groups (*p* < 0.05).

BMI	Absence shoulder pain (n = 206)	Mild shoulder pain (n = 78)	Moderate to intense shoulder pain (n = 108)	The highest VAS score (72 h after operation)
BMI < 18.5 (n = 25)	6	5	14	4.76 ± 1.35
18.5 ≤ BMI < 23 (n = 164)	64	26	74	4.94 ± 1.46
23 ≤ BMI < 30 (n = 165)	103	43	19	3.71 ± 1.48
BMI ≥ 30 (n = 38)	33	4	1	3.15 ± 1.51

## Discussion

**Figure 4:**
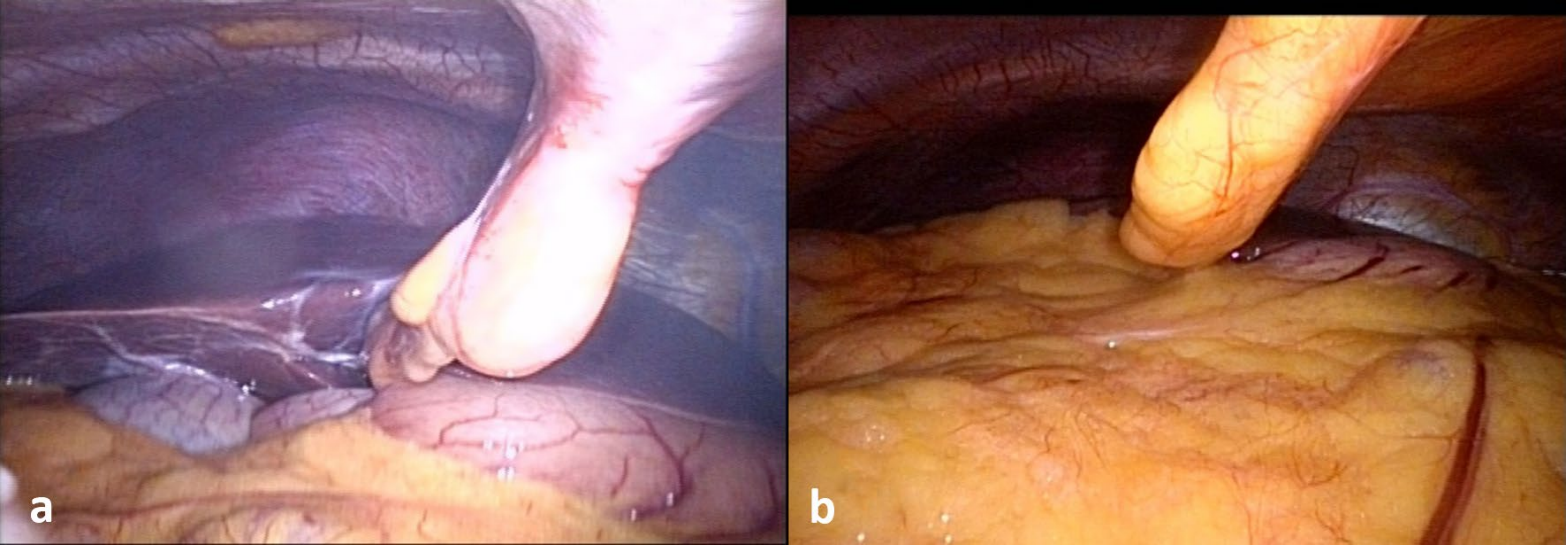
Upper abdominal imaging of two patients in the Trendelenburg position (30°). (**A**) 31Y, BMI = 19.3; (**B**) 32Y, BMI = 34.3.

PLSP is generally considered to develop due to diaphragmatic irritation from direct injury or stretching of CO2 gas^53^. This study found that BMI was negatively correlated with PLSP. When placing the patient in the Trendelenburg position, we could clearly observe the anatomical structures of the left and right lobes of the liver, the lower margin of the liver, the gallbladder and the diaphragm in thin patients. There is a large space in the upper abdomen. There may be a lot of gas trapped here after the operation. In obese patients, we can only observe the omentum since the rest of the anatomical structure is covered by the omentum, and the upper abdominal space is very small. Gas is unlikely to be retained here after surgery (Fig. 4). Previous imaging studies have found that thin patients are more likely to experience free gas in the abdominal cavity whether they are undergoing laparotomy or laparoscopic surgery^54,55^. The volume of the residual pneumoperitoneum is positively correlated with the intensity of shoulder pain after laparoscopy^53,56^. This is one explanation for why PLSP is more common in thin patients.

Our study found that PLSP in the vast majority of patients occurred on the first day after surgery. Previous studies have reported that after laparoscopic surgery, visceral pain predominates in the first 24 h but subsides from a peak soon after the operation, whereas shoulder pain, minor on the operative day, worsens and becomes significant on the following day^57^. In addition, PLSP is less responsive to analgesics than wound pain^21^. Proponents of pre-emptive analgesia believe that the most effective pre-emptive analgesia programmes are those that can limit the sensitization of the nervous system throughout the perioperative period^58,59^. Because the time characteristics of PLSP are different from abdominal pain^17,58^, if we do not consider the time characteristics of PLSP but treat PLSP according to the same plan used for the prevention and treatment of pain of the incision and viscera, the peak plasma concentration may not coincide with the peak PLSP. This may be one of the reasons why traditional interventions cannot effectively alleviate PLSP^59,60^.

Predicting the postoperative pain and/or analgesic needs of individual patients as accurately as possible will help us tailor-made personalized postoperative analgesia methods for each patient, which will certainly reduce the occurrence of severe postoperative pain. Thus, patients undergoing so-called minor surgery should be monitored more closely^15^. For the management of PLSP, we can comply with existing methods but change their timing or duration of administration^58^. This requires us to identify high-risk patients in advance and develop specific programmes according to the time characteristics of their occurrence. These need further study.

The main limitation of our study is that we did not measure the amount of carbon dioxide left in the abdominal cavity. Therefore, the correlation between the amount of residual carbon dioxide in the abdominal cavity and the severity of pain, as well as its correlation with BMI, is not clear.

## Conclusion

PLSP was negatively correlated with BMI. The occurrence of PLSP has obvious specific time characteristics. The results of the present study suggest that a series of studies according to the above clinical characteristics are required to explore measures to prevent or minimize shoulder pain resulting from this now well-standardized surgical procedure.
